# Strengthening microbiology laboratory capacity in Latin America and the Caribbean region to bolster global health security agenda

**DOI:** 10.1128/jcm.00032-26

**Published:** 2026-05-06

**Authors:** Wes Kim, Ana Da Costa, Andre Abreu, Amalia Corby

**Affiliations:** 1 Global Public Health Programs, American Society for Microbiology 11003, Washington, DC, USA; 2 General Coordination of Public Health Laboratories, Secretariat of Health Surveillance and Environment, Ministry of Health 42307, Brasília, Brazil; 3 Public Policy and Advocacy, American Society for Microbiology 11003, Washington, DC, USA

## EDITORIAL

The current global health ecosystem was devastated by funding cuts initiated by the United States and other high-income countries in 2025, while bracing for those anticipated for 2026 and beyond. According to a recent report, development assistance for health plummeted by 21% between 2024 and 2025 (from $49.6 billion to $39.1 billion), and further decline is expected over the next 5 years, dropping to $36.2 billion in 2030 (1). These reductions are, in part, due to refocusing domestic policy priorities with an emphasis on national security. Prime examples include the dissolution of major funding mechanisms from the United States and the United Kingdom aimed at combating antimicrobial resistance (AMR), which sent shockwaves across the AMR community. These funding streams included the U.S. Agency for International Development (established in 1961), which increased access to and appropriate use of life-saving antimicrobials and diagnostics, and the Fleming Fund (established in 2015), which increased AMR surveillance capabilities across 30 countries to inform appropriate use policies (2). Concurrently, the Global Fund fell short of its replenishment goals for 2025 (3). Although no single funder can solve the global AMR crisis, these collective decisions will have long-lasting and profoundly detrimental effects on global efforts to build the capacity needed to detect and prevent the spread of AMR.

Global health security needs to be a domestic policy priority. Communicable diseases, particularly those due to drug-resistant pathogens, are not bound by geographical borders, and neither should our efforts to combat them. The COVID-19 pandemic made us further appreciate the importance of public health infrastructure, particularly microbiology laboratories. At the same time, the pandemic exposed the vast inequities in access to vaccines, diagnostics, and personal protective equipment between high-income countries (HICs) and low- and middle-income countries (LMICs). These inequities, combined with our changing climate and the increasing globalization of people, products, and agricultural imports (4), will continue to increase risks of worldwide spread of communicable diseases. In light of these circumstances, funders and policymakers should double down on efforts to detect and prevent the spread of infectious disease and AMR by strengthening microbiology laboratory capacity for surveillance, increasing access and stewardship of drugs and diagnostics, building a robust pipeline of frontline workforce, and accelerating research and development of rapid diagnostics and new therapeutics and vaccines, through a multi-sectoral One Health approach. The recent divestments could not have been more poorly timed, as LMICs have recently made significant progress to surveil for and detect drug-resistant pathogens.

## CASE STUDY: LATIN AMERICA

Despite representing approximately 8% of the world population, Latin America reported 80 million COVID-19 cases and 1.7 million deaths, constituting 10% and 25% of global totals, respectively (5). Amid the 2022 Mpox resurgence, the Americas region accounted for 54% of all global confirmed cases, further underscoring the disproportionate burden (6). In addition, although a 2019 study noted that the relative AMR burden (counts and rates of deaths, years of life lost, disability-adjusted life years, and years lived with disability) in the Latin America and Caribbean (LAC) region was not as high as seen in sub-Saharan countries, a recent follow-up analysis forecasts LAC as a super-region with one of the highest all-age AMR mortality rates in 2050 (7, 8).

Health and population-related funding from external donors for LMICs, including the LAC region, increased between 2016 and 2022, with a particular surge during the COVID-19 pandemic (9). However, a closer look reveals a persistent gap in per capita funding between the LAC region and the rest of the LMICs (Table 1). This level of underfinancing leaves critical gaps in infectious disease surveillance and preparedness, creating conditions in which emerging pathogens can gain a foothold. While substantial investments into other LMICs’ health systems are warranted, the LAC region’s distinct challenges further underscore the need for more proportional and sustained investment. These challenges include recurrent and emerging epidemics, such as Mpox, Zika, and chikungunya, fragmented regional coordination mechanisms, and entrenched socioeconomic inequalities that hinder effective disease mitigation strategies (5). Additionally, globalization-driven mobility and environmental changes have increased the frequency and intensity of outbreaks in the region, further amplifying the vulnerability to future emerging pathogens and widening the region’s preparedness gap. Furthermore, within the LAC region, varying levels of capabilities and capacities necessitate country-tailored solutions to address these challenges (Table 2) (10).

**TABLE 1:** Comparative international health and population funding for Latin America and the Caribbean regions

Yr	All LMICs, excluding LAC region		LAC region	
	Total (USD millions)	Per capita	Total (USD millions)	Per capita
2016	$13,950	$2.53	$484	$0.80
2017	$13,429	$2.40	$453	$0.70
2018	$13,545	$2.39	$480	$0.80
2019	$12,298	$2.14	$368	$0.60
2020	$18,926	$3.26	$1,121	$1.80
2021	$23,959	$4.08	$1,533	$2.40
2022	$18,064	$3.05	$645	$1.00

**TABLE 2:** Comparison of LAC’s rankings on the Global Health Security Index

	Overall score	Prevention	Detection and reporting	Rapid response	Health system	Compliance with international norms	Risk environment
LAC average	37.7	27.4	28.1	38.8	30.1	46.6	55.0
Global average	38.9	28.4	32.3	37.6	31.5	47.8	55.8
LAC range	20.9–57.0	5.3–50.9	4.2–58.1	22.1–64.8	9.1–71.7	26.0–68.1	34.4–73.6

The American Society for Microbiology’s (ASM) initiative to strengthen surveillance of drug-resistant *Bordetella pertussis* in Brazil, Mexico, and Peru, supported by funding from the US Centers for Disease Control and Prevention (US CDC) Global Antimicrobial Resistance Laboratory Network, exemplifies critical investment and efforts to enhance global health security (11). Pertussis, or whooping cough, surged in parts of the United States in 2024, and incidence has remained elevated throughout 2025 (12–14). Similarly, outbreaks were also reported in Europe (15, 16), Asia, and Latin America, including Brazil, Mexico, and Peru, within this same time period (17, 18). The Americas region reported a substantial increase in the number of cases, rising from 4,139 in 2023 to 43,751 cases in 2024 (18). In 2025, pertussis incidence in Latin America continued to climb, associated with a concerning rise in mortality reported throughout the first 19 epidemiological weeks, with Brazil reporting 1,634 confirmed cases and 5 deaths, and Mexico documenting 943 cases and 51 deaths (18). Peru reported 404 confirmed and 219 probable cases, associated with 13 deaths (18). These numbers continue to increase, with disproportionate impact on infants and young children, despite relatively high vaccination rates. These findings highlight that pertussis burden and mortality vary considerably across countries in the region, reflecting heterogeneous epidemiologic contexts and underscoring the need for tailored evaluation and support strategies.

As part of an ASM-led initiative in collaboration with Instituto Adolfo Lutz (Brazil), the Instituto de Diagnóstico y Referencia Epidemiológicos “Dr. Manuel Martínez Báez” (InDRE, Mexico), and the US CDC, a recent retrospective analysis of macrolide susceptibility (erythromycin, azithromycin, and clarithromycin) in *Bordetella pertussis* in archived clinical isolates from Brazil and Mexico (2004−2022) found no evidence of resistance (S. Hatoum, E. Montilla‑Escudero, V. Cantarelli, V. Silva, M. Urrego, M. Pierre, L.A. Sapian, E. González Durán, C. Damián Peralta, C.H. Bäcker, N.A. Montes Colima, R. Aparicio Antonio, I. López‑Martínez, D. Leite, R. Polatto, A. Bertani, L. Santos, A.L. de Abreu, G. Andrade Pereira, J. Stellinge, I. McCall, P.C. Callaway, J. Clemente, L.C. Pawloski, W. Kim, A.R. Da Costa, unpublished data). However, in 2024, the first documented case of macrolide-resistant *Bordetella pertussis* in the LAC region was reported in Mexico (19). That same year, Brazil also reported the first 18 macrolide-resistant cases, underscoring the emergence of resistance in the region (20). In 2025, Peru reported nearly 57% macrolide-resistant *B. pertussis* cases (21).

Since 2021, ASM has partnered with national and state reference laboratories in Brazil, Mexico, and Peru to detect circulating drug-resistant strains of *B. pertussis* through enhanced surveillance and effective data sharing. To date, 3 national reference laboratories have adopted standardized procedures for macrolide susceptibility testing, and 12 state laboratories have strengthened diagnostic workflows, including sample processing and data transportation, to support timely identification and reporting of *B. pertussis* cases. Our work includes the provision of training and standardized tools (e.g., job aides) for identifying infections and conducting antibiotic susceptibility tests; inter-country knowledge sharing; enrollment in external quality assessments to ensure epidemiological data are accurate and better inform public health policy; and design of an LAC-based coordinating center to sustain investments by the US CDC’s Global Antimicrobial Resistance Laboratory Network. This work has the potential to sustainably detect and control macrolide-resistant *B. pertussis* across LAC.

## THE CASE FOR CONTINUED INVESTMENT

World Health Assembly Resolution (WHA76.5, 2023) calls for strengthening global diagnostics capacities as a foundation for improving overall healthcare (22). The current trend of HICs divesting from health system strengthening, particularly for microbiology laboratories, is detrimental to global health.

The US government is implementing a new “America First Global Health Strategy” (23), citing significant, longstanding issues within foreign assistance programs as the impetus for pulling back investments. Although the strategy relies heavily on bilateral agreements with partner governments, it is unclear if the United States will invest in communicable diseases at prior levels. Programs such as the US CDC’s Global Antimicrobial Resistance Laboratory Network are critical for building coordinated global capacity to detect antimicrobial resistance and support prevention strategies across countries, and coordinated efforts like the US CDC’s Transatlantic Taskforce on Antimicrobial Resistance provide avenues for donor countries to collaborate for greater impact. These collective efforts enable countries to become better prepared to rapidly and effectively address emerging pathogens before they cause catastrophes and once again paralyze the globe.

As governments in the LAC region commit to investing more in their health systems, global health resiliency is bolstered. While each country must prioritize and take ownership of its health security, many do not have the resources to do so. HICs’ support for programs that create sustainable investment stewardship in LMICs is still essential, as is health diplomacy to ensure recipient buy-in and a commitment to taking ownership. In countries like Brazil, external support has proven fundamental for implementing strategies to prevent and control AMR and for formulating public policies within the country’s Unified Health System. Given the decreased funding ecosystem, we need to continue maximizing each dollar spent on strengthening health systems. This can be done through improved, rapid testing, more peer-to-peer knowledge exchange between LMICs (i.e., expanding on the training-of-trainers model), and reduced redundancies due to siloed/vertical programs by optimizing existing diagnostic infrastructure and expanding shared services. Utilizing existing collaboration mechanisms, particularly the Pan American Health Organization’s Collaborating Centers, could be an efficient approach (24).

Finally, as changing regional climates spur new human and animal migration patterns, increasing the potential for novel human-animal interfaces and zoonoses, a One Health strategy that integrates multisectoral surveillance of zoonotic diseases should guide investments. Zoonoses are responsible for more than 6 out of every 10 known infectious diseases in people, and 3 out of every 4 new or emerging infectious diseases in people (25). Therefore, in addition to human surveillance, we also need both animal and environmental surveillance, with all three integrated into programs.

With any emerging diseases and pandemics, the ability to rapidly, accurately surveil and diagnose is key for prevention and control responses, particularly in a manner that enables equitable access to diagnostic services. The COVID-19 pandemic placed a spotlight on the world’s inadequacies in addressing a major global health disaster. We cannot continue to make the same mistakes.
